# Genome-Wide Association Studies for Milk Somatic Cell Score in Romanian Dairy Cattle

**DOI:** 10.3390/genes12101495

**Published:** 2021-09-24

**Authors:** Daniela Elena Ilie, Alexandru Eugeniu Mizeranschi, Ciprian Valentin Mihali, Radu Ionel Neamț, George Vlad Goilean, Ovidiu Ionuț Georgescu, Daniela Zaharie, Mihai Carabaș, Ioan Huțu

**Affiliations:** 1 The Molecular Research Department, Research and Development Station for Bovine Arad, Bodrogului Street, No. 32, 310059 Arad, Romania; alex.mizeranschi@gmail.com (A.E.M.); mihaliciprian@yahoo.com (C.V.M.); neamtr@yahoo.com (R.I.N.); vladgoilean@yahoo.com (G.V.G.); 2 Faculty of Veterinary Medicine, Banat’s University of Agricultural Sciences and Veterinary Medicine “King Michael I of Romania” from Timisoara, Calea Aradului No. 119, 300645 Timisoara, Romania; georgescu_ovidiu.ionut@yahoo.com (O.I.G.); ioan.hutu@fmvt.ro (I.H.); 3 Faculty of Mathematics and Computer Science, West University of Timișoara, 300223 Timisoara, Romania; daniela.zaharie@e-uvt.ro; 4 Faculty of Automatic Control and Computer Science, Politehnica University of Bucharest, 060042 București, Romania; mihai.carabas@roedu.net

**Keywords:** genome-wide association study (GWAS), mastitis, Romanian cattle, somatic cell score

## Abstract

Mastitis is one of the most frequently encountered diseases in dairy cattle, negatively affecting animal welfare and milk production. For this reason, contributions to understanding its genomic architecture are of great interest. Genome-wide association studies (GWAS) have identified multiple loci associated with somatic cell score (SCS) and mastitis in cattle. However, most of the studies have been conducted in different parts of the world on various breeds, and none of the investigations have studied the genetic architecture of mastitis in Romanian dairy cattle breeds up to this point in time. In this study, we report the first GWAS for SCS in dairy cattle breeds from Romania. For GWAS, we used an Axiom Bovine v3 SNP-chip (>63,000 Single Nucleotide Polymorphism -SNPs) and 33,330 records from 690 cows belonging to Romanian Spotted (RS) and Romanian Brown (RB) cattle. The results found one SNP significantly associated with SCS in the RS breed and 40 suggestive SNPs with −log_10_ (*p*) from 4 to 4.9 for RS and from 4 to 5.4 in RB. From these, 14 markers were located near 12 known genes (*AKAP8*, *CLHC1*, *MEGF10*, *SATB2*, *GATA6*, *SPATA6*, *COL12A1*, *EPS8*, *LUZP2*, *RAMAC*, *IL12A* and *ANKRD55)* in RB cattle, 3 markers were close to *ZDHHC19*, *DAPK1* and *MMP7* genes, while one SNP overlapped the *HERC3* gene in RS cattle. Four genes (*HERC3*, *LUZP2*, *AKAP8* and *MEGF10*) associated with SCS in this study were previously reported in different studies. The most significant SNP (rs110749552) associated with SCS was located within the *HERC3* gene. In both breeds, the SNPs and position of association signals were distinct among the three parities, denoting that mastitis is controlled by different genes that are dependent according to parity. The current results contribute to an expansion in the body of knowledge regarding the proportion of genetic variability explained by SNPs for SCS in dairy cattle.

## 1. Introduction

In recent decades, the prevalence of clinical mastitis in cattle has increased significantly [1] in most of developed agricultural countries where farming practices are more intensive. This tendency is manifested in parallel with a higher selection pressure for high milk yields in modern dairy breeds. Therefore, improving the understatement of the genetic base of mastitis and its indicator traits represents a major goal for the dairy cattle breeding industry as it can improve udder health and increase the milk quality, while also reducing early involuntary culling, discarded milk, veterinary services and labor costs.

In dairy cattle, many studies have been conducted in different parts of the world on various breeds in order to investigate the genetic base of mastitis. Research results from more than two decades have shown the association between the major histocompatibility complex (MHC) and susceptibility or resistance to intramammary infection [2]. In the beginning, most of the studies focused on MHC genes, particularly the DRB region, for which various associations between allelic variants and immune response and mastitis resistance have been reported by several authors [3,4,5,6]. Later, quantitative trait loci (QTL) responsible for clinical mastitis and somatic cell counts (SCC) have been identified in the majority of the chromosomes. Among these, QTL on chromosome 1, 3, 7, 8, 14, 18, 21 and 23 were confirmed in different separate studies [1]. Moreover, the technological developments of molecular genetics from the last decade, followed by sequencing of the *Bos taurus* genome in 2009 [7], enabled the identification of several molecular markers responsible for susceptibility or resistance to mastitis. The appearance of high-throughput genotyping technologies allowed genome-wide association studies to be carried out in cattle [8]. By the application of new genetic analysis methods and genome-wide association studies (GWAS), exploring the genetic architecture of the important traits in different cattle breeds [9] and detecting the significant single nucleotide polymorphisms (SNPs) or genomic regions associated with mastitis in dairy cattle became possible. For instance, Meredith et al. [8] used 773 Holstein-Friesian AI sires with progeny genotyped by using the Illumina BovineSNP50 Genotyping Beadchip and identified nine SNPs that were significantly associated with somatic cell score (SCS), of which three were located on chromosomes 6 and 10 within known QTL regions for SCS, and six SNPs placed outside known QTL regions for SCS were located on chromosomes 6, 15 and 20. One year later, the same author [10], by using 702 Holstein-Friesian sires that were genotyped for 777,962 SNPs on the Illumina High-density Bovine BeadChip, detected 28 QTL regions associated with SCS, with 138 SNPs located across 15 chromosomes (1, 3, 4, 5, 6, 9, 10, 13, 17, 20, 21, 22, 23, 24, 25 and 26). Strillacci et al. [11] conducted a GWAS for SCS in Valdostana Red Pied cattle and found genes involved in mastitis resistance or variation of SCS in QTL on chromosomes 9, 13, 15, 17, 19, 21 and 22. Another study [12] analyzed 544 Holstein and Holstein × Jersey cows and identified six SNPs on chromosomes 1, 5, 10, 18 and 26 that were associated with traits derived from SCC. Recently, a genome-wide association study [9] found three significant SNPs on chromosomes 5, 8 and 22 that were associated with SCS in 2410 Xinjiang Brown cows. According to the current release of the Cattle Quantitative Trait Loci (QTL) Database of Animal QTLdb (The Animal Quantitative Trait Loci Database, https://www.animalgenome.org/cgi-bin/QTLdb/BT/index, accessed on 20 May 2021 [13]), a total of 2401 QTLs, spread on most bovine chromosomes, were found to be associated with mastitis.

Mastitis resistance is a complex trait and although important progress has been made in this area, the research efforts should be intensified in order to better understand the genetic architecture of this important trait and, also, to find new possible markers in order to improve the possibilities of genetic progress and enable more accurate selection against mastitis. Moreover, although GWAS was widely used to identify the genetic variants associated with mastitis and its indicator traits such as SCS, to the best of our knowledge, none of the studies investigated the genetic architecture of SCS in Romanian dairy cattle breeds. To address this issue, in the current study, we used the GWAS approach in 723 Romanian Spotted (RS, national name: Baltata Romaneasca) breed (Breeds of Livestock, Department of Animal Science; Breeds of Livestock—Baltata Romaneasca Cattle (http://afs.okstate.edu/breeds/cattle/baltataromaneasca/index.html/, accessed on 25 August 2021 [14]) and Romanian Brown (BR, national name Bruna de Maramures) cattle breeds from three distinct herds to identify candidate genes and genomic regions associated with SCS.

## 2. Materials and Methods

### 2.1. Animals and Phenotypes

This study was conducted on 723 dairy cattle out of which 601 were RS and 122 were RB cattle. The animals involved were managed in three breeding herds: two experimental herds belonging to the Romanian Academy of Agricultural Science (the Research and Development Station for Bovine Arad and the Research and Development Station for Bovine Sighetu Marmatiei) and one commercial herd located in Berliste. All cows involved in the study were kept under comparable housing and feeding conditions, with similar milking and sanitary conditions, and were mechanically milked twice a day. All the animals had at least 3 monthly test-day records per lactation. Only data corresponding to the first three lactations were included in the analysis.

Phenotypic data consisted of 33,330 SCC records, of which 24,295 were for RS and 9035 were for RB cattle. For SCC determination, milk records were conducted every 28 days by the Official Dairy Control service. The milk samples were analyzed for SCC determination in the laboratory of the Milk Quality Control Foundation (Cluj-Napoca, Romania) by using CombiFoss™ FT + integrating MilkoScan™ FT + and Fossomatic™ FC.

In order to achieve a normal distribution of the data, the values of SCC were transformed to SCS according to the formula of Wiggans and Shook [15] as *SCS = log2 (SCC/100,000) + 3*, specified by the international standard (Interbull Code of Practice. https://interbull.org/ib/codeofpractice, accessed on 25 August 2021 [16]).

### 2.2. Sampling and Genotyping

Biological samples needed for genotyping consisted of whole blood and hair roots that were collected by trained veterinarians. Blood samples (*n* = 627) were collected from the tail vein in vacutainers containing K3EDTA as an anticoagulant, and the hair roots (approximately 20–40 hairs with follicles attached) were collected from the tail of the animals (*n* = 96). All protocols used for collection of the biological samples were approved by the Ethics Committee of the Research and Development Station for Bovine Arad (Statement no 88/04.10.2019). After collection, the samples were transferred to the laboratory and stored at 4 °C, and they were subsequently sent to IFN Schönow GmbH (Bernau bei Berlin, Germany) for DNA extraction and genotyping using an Axiom Bovine v3 microarray (based on the reference genome Bos_taurus_UMD_3.1.1), which includes >63,000 SNPs. The used SNP chip included a high percentage of SNPs that are found distributed on all chromosomes. The SNPs on the array include 44,000 markers from the Council on Dairy Cattle Breeding (CDCB), International Society for Animal Genetics (ISAG) core parentage markers, VIP trait-associated SNPs and SNPs optimized for STR imputation. In total, 723 samples were genotyped.

### 2.3. Quality Control (QC)

Data quality control was performed in order to remove SNPs and individuals with insufficient genotyping quality. QC procedures were carried out by using PLINK v1.90b6.16 64-bit [17]. The SNPs located on the sex chromosome or those with unknown positions were removed from the current study. Individuals with a call rate of less than 95% were excluded. The SNPs with call rates <95% or with minor allele frequency (MAF) < 0.05 and SNPs with genotypes not in accordance with the Hardy–Weinberg equilibrium (*p* > 10^−6^) were eliminated.

### 2.4. Association Analysis

Principal component analysis (PCA) was performed for each breed-specific subpopulation in order to check for population stratification. In both cases, the first 20 principal components were computed by using the R package argyle v0.2.2 [18]. Based on the proportion of variation explained by principal components, we noted that the first 8 principal components explained the most significant amount of genetic variability (23.86% of the total variance in RB and 17.49% in RS, respectively) and, thus, captured any possible effects of population stratification (Supplementary Figure S1). For this reason, we included the first 8 principal components into the GWAS study as covariates for both breeds.

The GWAS was performed for the SCS trait by using two-stage analysis. First, a random-regression test-day model using only phenotypes and pedigree data was implemented in Blupf90 [19] by using the combined variable herd-year-season as the fixed effect in order to represent contemporary groups and by using fourth-order Legendre polynomial coefficients for both fixed and random effects (additive genetic and permanent environmental effects) in order to represent components of the lactation curve. The variance components were computed by using the *remlf90* command, after which the estimated breeding values (EBVs) and prediction error variances and covariances (PEVs) were computed using the *blupf90* command with the *store_pev_pec* option. PEVs were used to compute EBV reliabilities by using the following formula:

*Rel* = 1 − *PEV/V_A_*, (1)

where *V_A_* is the additive genetic variance.

EBV reliabilities were then used to compute de-regressed proofs (DRPs) by using the R package DRP [20], which implements the approach proposed by Garrick et al. [21]. More specifically, the *wideDRP* function was used by setting the heritability to its estimated value and using the default value of 0.5 for the c parameter, which represents the fraction of genetic variance not explained by markers.

For the second GWAS stage, DRPs were used as phenotypes in the rrBLUP package v.4.6.1 [22] by using the *GWAS* function, with only the first 8 principal components included as fixed effects. SNP effects were computed within the same R package by using the *mixed.solve* function. A genome-wide significance threshold of *p* < 1.00 × 10^−4^ was used, and SNPs exceeding this threshold were considered as candidate variants. Manhattan plots were created by using the R package qqman v.0.1.4 [23].

Linkage disequilibrium analyses for some of the significant SNPs on chromosome 7 and 9 in RB breed and chromosome 6 in RS breed and SNPs from the surrounding regions (±500,000 KBp relative to the SNP’s position) were performed by using the *IntRegionalPlot* function of the R package IntAssoPlot v.0.99.21 [24]. This function produces a figure consisting of two layers. The top layer represents a scatter plot of the −log_10_ (*p*) values for all the SNPs in a selected region, with the chromosomal position on the x-axis. The bottom layer depicts a heat map of LD blocks for each of the pairs of SNPs within the region. In addition, the function adds lines between the two layers to show which SNP corresponds to which LD block.

Finally, significant SNPs were annotated with overlapped and downstream/upstream genes within a ± 1 MBp range (2 MBp total) by using the R package VariantAnnotation v.1.28.13 [25], and annotations were obtained via the R package GenomicFeatures v.1.34.8 [26] from the UMD3.1.94 GTF file downloaded from Ensembl.

## 3. Results

### 3.1. Descriptive Statistics

A total number of 690 cows with 33,330 test-day milk records were used in this study. The number of records for second (31.66%) and third parity (24.71%) was lower than those for the first lactation (43.63%). For the two breed-specific subpopulations by parity (L_1_–L_3_), the corresponding percentages were as follows: 45.61%, 30.78% and 23.61% in RS and 38.32%, 34.00% and 27.68% in the RB breed, respectively. The descriptive statistics of the observed phenotypes for each breed and lactation are presented in Table 1.

According to our results, the average SCSs among parities (L_1_–L_3_) were 2.81, 3.06 and 3.27 in RS and 4.53, 4.51 and 4.62 in RB cattle, respectively. The maximum level of SCS was 8.84; nevertheless, the number of cows with this level of SCS was low in both breeds. The overall mean SCS for RS was 2.99 ± 1.82 and 4.55 ± 1.89 in RB cattle.

Estimates of heritability for the SCS showed different variation between breeds over the 305 days of lactation. The range of SCS heritability values for L_1_–L_3_ in RS (0.08, 0.10 and 0.11) was higher than compared to RB (0.03, 0.07 and 0.06). The highest heritability was recorded for RS cattle in the third lactation (0.11). Between breeds, a distinct pattern of variation in heritability values was observed. The shape of the SCS heritability curves (Supplementary Figure S2) was similar for L_1_–L_3_ in the RS breed, with the highest values in the first and last parts of the lactation. Conversely, the analysis of daily SCS heritabilities in the RB breed revealed different shapes of curves between the first, second and third lactation. This could be due to the smaller number of RB animals included in the study, which means that possible outlier individuals had a larger impact on the breed-specific population average. The SCS heritabilities in RB decreased in the first part of the lactation (up to 110 days in milk) and then remained on the plateau in mid-lactation (up to 200 days in milk), after which it gradually increased in the last part of the lactation (up to 305 days in milk). This trend appears similar to that of the incidence of mastitis.

### 3.2. Quality Control

After computing descriptive statistics for the phenotypic data, quality control checking of genotyping data was the second step in our data analysis. Since the success of a genome-wide association study is considerably influenced by appropriate study design and data analysis workflow [27], extra attention was paid towards data quality control of genotyping data. After eliminating markers from unplaced contigs and sex chromosomes, markers with unknown chromosomes or positions within the chromosome, multiallelic markers, indels and duplicate SNPs from the original loci of the microarray, a number of 59,915 markers for 723 animals entered the QC phase. From these, a number of animals and SNPs were discarded as follows: 11 animals that had greater than 5% missing SNP calls; 795 SNPs with call rates <95%; 17,701 SNPs with MAF <0.05; and 1114 SNPs with genotypes that are not in accordance with the Hardy–Weinberg equilibrium (*p* > 10^−6^). Finally, after the quality control analysis, a data set of 40,305 SNPs across the entire bovine genome from 690 cows (571 RS and 119 RB) was used in the subsequent analysis to carry out genotype association tests.

### 3.3. Principal Component Analysis

**Figure 1:**
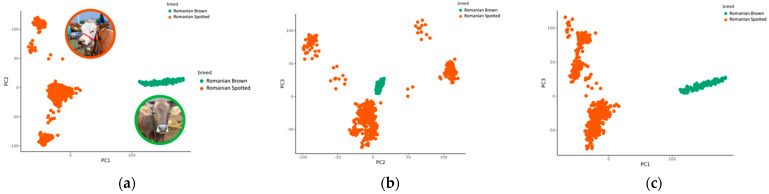
Population structure from the principal component analysis of the 40,305 single nucleotide polymorphisms (SNPs) and 690 cattle. Population structure is presented as pairwise scatter plots (**a**–**c**) of the first three principal components (PC), with green and orange dots representing the two breeds (Romanian Spotted in orange and Romanian Brown in green). (**a**) PC1 vs. PC2; (**b**) PC2 vs. PC3; (**c**) PC1 vs. PC3.

The animals involved in this study came from two breeds and three breeding herds. Thus, in order to assess population stratification, we performed principal component analysis. The PCA plot revealed a clear population structure for the animals in the two cattle breeds included in the study, showing a clear separation between individuals, according to breed (Figure 1). Clusters of the same color represent individuals from the same breed. All animals from the RB breed clustered together in all pairwise scatter plots of the first three principal components, but they were separated from the RS individuals. From the three plots in Figure 1, we observed that PC1 separates the individuals from the two breeds, whereas PC2 and PC3 divide individuals within the RS breed. The individuals from the RS breed split into several subgroups that were best observed in the scatterplot of PC2 and PC3 (Figure 1b). We have conducted a separate investigation into the origins of these different clusters (data not shown) and concluded that one subgroup consists of cows with paternity from one single sire and the rest of the subgroups consist of cows from several different sires.

### 3.4. Significant SNPs Associated with SCS

**Figure 2:**
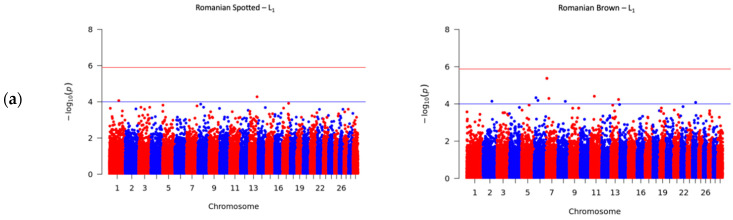

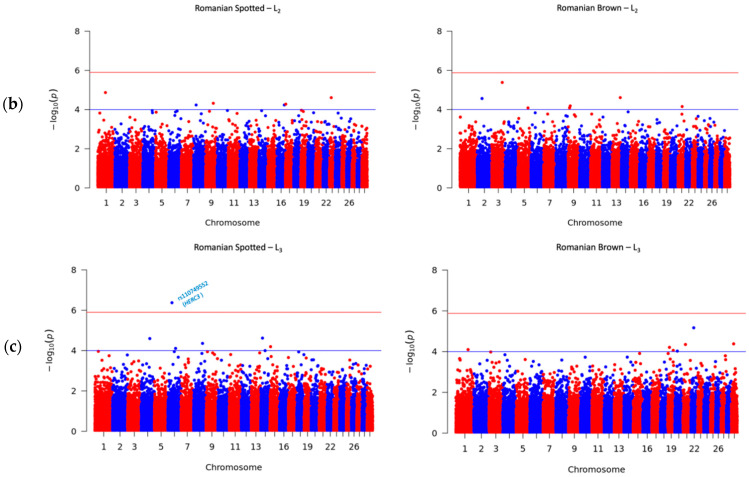
Manhattan plots for somatic cell score (SCS) in the Romanian Spotted (**left**) and Romanian Brown breed (**right**). The blue line indicates the suggestive *p* value threshold of −log_10_ (*p*) ≥ 4.00. The red line indicates the Bonferroni genome-wide significance *p* value threshold at −log_10_ (*p*) ≥ 5.89. The *y*-axis shows the −log_10_ (*p*) of 40,305 SNPs, and the *x*-axis shows the chromosomal positions. (**a**) First lactation; (**b**) Second lactation; (**c**) Third lactation.

To investigate the genetic variation that underlies the SCS, GWAS was performed in roder to identify the associated SNP loci in cattle. The Manhattan plots for SCS in the RS and RB breeds are shown in Figure 2. The upper horizontal lines represent the Bonferroni-adjusted genome-wide significance threshold −log_10_ (*p*) ≥ 5.89, and the inferior horizontal lines represent the suggestive threshold of −log_10_ (*p*) ≥ 4.00, which was set to report further significant associations that were not observed under Bonferroni corrections. The suggestive threshold was chosen according to previous studies where researchers defined a suggestive threshold between *p* < 10^−5^ and *p* < 10^−3^ [28,29]. The candidate genes were detected by verifying whether significant or suggestive SNPs overlapped a gene or were located within 1 MBp upstream or downstream from a gene. The genomic positions were based on the UMD3.1 genome assembly of *Bos taurus* [7].

The genome-wide SNPs detected for SCS among parities (L_1_–L_3_) are presented in Table 2 for the RS breed and Table 3 for the RB breed. A total number of 41 SNPs were detected in the RS and RB breeds, of which 40 SNPs passed the suggestive threshold and, ultimately, one SNP passed the significance threshold after Bonferroni correction and was associated with SCS in the RS breed in L_3_ (Figure 2c, left side). As shown in Table 2, for the RS breed, 3 out of 15 SNPs were located near three known genes, and one SNP overlapped the *HERC3* gene. Of the fifteen SNPs in the RS breed, AX-106761943 (rs110749552) was located on chromosome 6 within the *HERC3* gene (HECT and RLD domain containing E3 ubiquitin protein ligase 3) and was the most significant SNP for SCS (log_10_ (*p*) = 6.37). In the RB breed, the genome-wide analysis detected 26 SNPs that reached genome-wide significance for association with SCS (Table 3). Among these SNPs, 14 out of 26 SNPs were located near 12 known genes; two SNPs, AX-106741653 and AX-115114947, were located near the *AKAP8* gene, and two other SNPs, AX-106735825 (rs43585636) and AX-117085949 (rs43585209) were located near the *COL12A1* gene, respectively. None of the 15 detected SNPs in the RS breed were in common with the detected 26 SNPs that reached genome-wide significance for association with SCS in the RB breed.

### 3.5. Linkage Disequilibrium (LD) Blocks of the Significant SNPs

**Figure 3:**
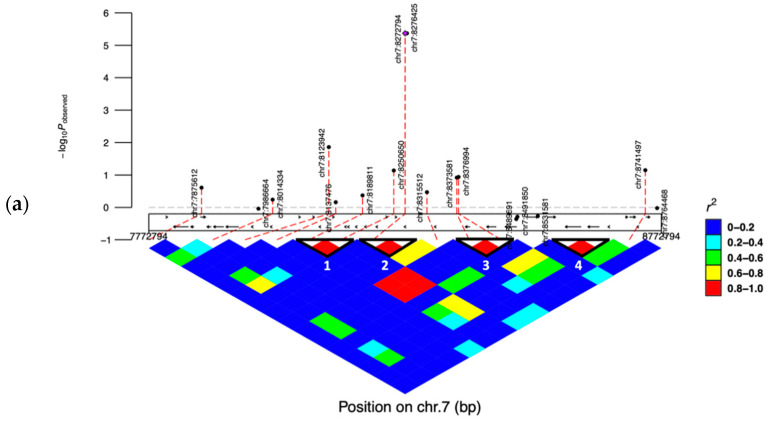

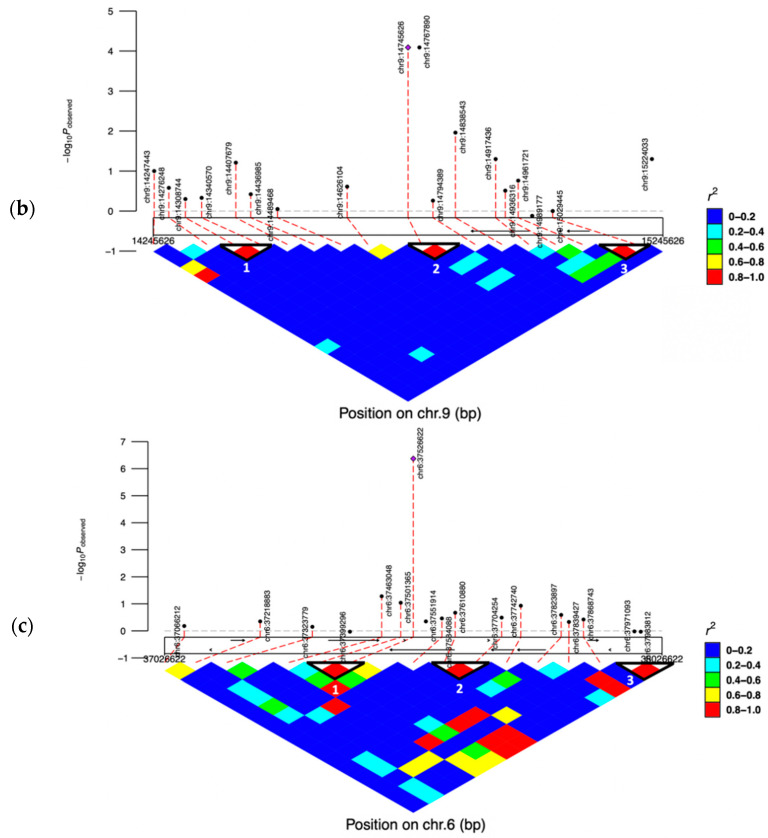
Linkage disequilibrium (LD) analysis. Solid line triangles refer to blocks of high linkage disequilibrium. The color of one square refers to the LD level (*r*^2^) between two SNPs. (**a**) LD pattern for 17 SNPs in the region between 7,772,794 to 8,772,794 Bp on chromosome 7; (**b**) LD pattern for 20 SNPs in the region between 14,245,626 to 15,245,626 Bp on chromosome 9; (**c**) LD pattern for 17 SNPs in the region between 37,026,622 to 38,026,622 Bp on chromosome 6.

We performed the linkage disequilibrium analysis (*r*^2^ value) on chromosome 7 in the RB breed for a region of 1 MBp interval (from 7,772,794 to 8,772,794 Bp), which covers seventeen SNPs (Figure 3a). High LD blocks were considered for SNPs with *r*^2^ ≥ 0.8. Four high LD blocks were revealed where the most significant SNP (AX-106741653) was located in a block along with another significant SNP (AX-115114947). The two significant SNPs in block 2 were located upstream of the *AKAP8* gene at distances of 514,640 and 511,009 Bp, respectively. A second linkage disequilibrium analysis for a total of twenty SNPs on chromosome 9 in the RB breed was performed and showed three LD blocks (Figure 3b), each of them including two SNPs, of which the SNPs in block 2 were located upstream of the *COL12A1* gene. For the RS breed, we performed a single LD analysis on chromosome 6 in a region of ±500,000 KBp relative to the position 37,526,622 bp of the SNP AX-106761943 (rs110749552), which was the only marker in our study that had a significant association according to Bonferroni correction. For the RS breed, the LD analysis revealed three high LD blocks (Figure 3c), of which the first block contained the previously mentioned SNP AX-106761943 and a non-significant SNP AX-117085324 (rs109478631), both of them located within the *HERC3* gene.

## 4. Discussion

Since mastitis is one of the most frequent diseases in dairy cattle negatively affecting milk production and animal welfare, contributions to understanding the genomic architecture are of great interest. Mastitis, on the other hand, is exceedingly difficult to genetically manage due to its low heritability. However, as a result of the genetic correlation between mastitis incidence and SCC, the latter indicator trait has been used as an indirect technique for predicting udder infections [30]. Moreover, SCC is recorded in most cases through the Official Dairy Control service in all developed countries and is used as a marker to monitor the prevalence of mastitis in dairy cattle [31], thus being more accessible to farmers compared to microbiological tests. In the present study we have performed GWAS and detected SNP variants associated with SCS variation in native Romanian dairy cattle breeds. To our knowledge, this is the first research study that identified potential causative genes and reported associated SNP variants linked to mastitis in Romanian cattle breeds.

In our study, the overall mean ± SD SCS values for the RS and RB breeds were 2.99 ± 1.82 and 4.55 ± 1.89, respectively. These averages are in agreement with previous studies. For instance, Alam et al. [32] reported means of SCS for five lactations of Holstein cows to range between 3.50 and 4.32. Zavadilová et al. [33], using records from the first three lactations of Fleckvieh cows, reported mean SCS of 3.16 to 4.01. The average SCS in this study increased for later lactations, and the trends were similar for both breeds, with the lowest values of SCS during the first and second lactation and higher values during the third lactation. The increasing trend of SCS with the increment of lactation rank observed in our study was also reported in other articles [32,34,35]. Differences in SCS suggest that increased milk flow may contribute to an increase in SCS for later lactations.

The average heritabilities for SCS over the entire days-in-milk range for 305 days were small to moderate and varied from 0.03 (RB) to 0.11 (RS), which are in agreement with previous studies. For example, for the Simmental breed, Wei et al. [36] reported values for SCS heritability of 0.09, whereas Mancin et al. [37] found a slightly higher heritability of 0.133 in Alpine Grey cattle, a local dual-purpose breed from the Italian Alps. However, substantial variations in the heritability of SCS were reported in different studies for distinct breeds. Previous estimates of heritability were low in Jersey and Holstein cattle (0.01–0.06) [38,39], moderate in Finnish Ayrshire (0.07–0.12) [40] and Valdostana cattle (0.06) [41] and higher values in Holstein-Friesian cattle were estimated (0.19–0.21) [42,43]. Various studies have resulted in the conclusion that several factors, including populations, statistical models and estimation methods, can account for these discrepancies in heritabilities [33,44].

Genome-wide association studies provide a valuable source of information for the comprehension of genetic mechanisms that point out the various phenotypes in dairy cattle. Nevertheless, when looking at the GWAS results so far, it is clear that identifying specific SNPs and genes located around them linked to SCS is difficult. As observed in the present study (Table 2 and Table 3), from a total of 41 SNPs that were found to be associated with SCS in both Romanian cattle breeds, the majority of them were dissimilar from significant SNPs reported in previous publications [8,10,11,29,45,46], while the *HERC3*, *LUZP2*, *AKAP8* and *MEGF10* genes located near significant SNPs on chromosome 6, 29 and 7 are consistent with other studies [47,48,49], which strongly suggests that these genes are candidate genes for SCS.

In the RS breed, the GWAS results pointed out 15 SNPs associated with SCS. Out of the significant SNPs, three markers were close to the *ZDHHC19*, *DAPK1* and *MMP7* genes, while one SNP overlapped the *HERC3* gene. The most significant SNP in our study, rs110749552, (6:g.36100172G > A on ARS-UCD1.2; −log_10_ (*p*) = 6.36) associated with SCS in RS breed in L_3_ was located within the *HERC3* gene that encodes a member of the HERC ubiquitin ligase family. Significant associations of the *HERC3* gene with different traits have been previously reported by several authors in cattle or sheep populations. This gene was previously found to be related to mastitis and somatic cell count in Ethiopian cattle populations [47]. According to Jiang [50], the *HERC3* gene was associated with milk protein percentage in Chinese Holstein cattle. In French Holstein, the *HERC3* gene had highly significant effects on αs1-casein [51]. Porto-Neto et al. [52] explored the genetic architecture of ten traits in tropical cattle breeds and reported the *HERC3* as a candidate gene for yearling weight. In addition, Al Kalaldeh [53] showed that the *HERC3* gene on *Ovis aries* chromosome 6 fell within the QTL region that underlies genetic variation for parasite resistance in sheep and also noticed that the genes from the HERC family of ubiquitin ligases are associated with antigen processing: ubiquitination and proteasome degradation; immune system; class I MHC mediated antigen processing and presentation; and adaptive immune system pathways. However, this is the second study where this gene has been found to be involved in the genetic makeup of mastitis.

With regards to the RB breed, the GWAS results revealed 26 promising SNPs associated with SCS. Out of the significant SNPs, 14 were located near 12 known genes, including *AKAP8*, *CLHC1*, *MEGF10*, *SATB2*, *GATA6*, *SPATA6*, *COL12A1*, *EPS8*, *LUZP2*, *RAMAC*, *IL12A* and *ANKRD55.* On chromosome 7, the rs42228650 SNP was close to *MEGF10*, a gene that was found to be associated with SCS in dairy cows in a previous investigation [48] and was considered as a candidate gene associated with residuals for fecal egg counts of gastrointestinal nematodes in German Black Pied cattle in a recent study [54]. On the same chromosome, two significant SNPs (AX-106741653 and AX-115114947) in the RB breed were located close to the *AKAP8* gene, which was associated with somatic cell count in seven different populations of dairy cows in a complex study that used the combined approach of examining previous results from differential gene expression analysis and GWAS studies to identify the key pathways and candidate genes that potentially confer genetic resistance to mastitis in dairy cows [48]. Furthermore, another relevant gene revealed in our work located 519,687 Bp from the rs110955314 SNP was *LUZP2* on chromosome 29, which has been associated with SCS [48] and milk fatty acid traits in cattle [55].

In addition to the genes known to be associated with mastitis, the present study identified novel candidate genes associated with SCS in Romanian cattle. Some of these genes have been shown to be linked with other traits in ruminants. For instance, the rs109189476 SNP on chromosome 2 was close to *SATB2*, a gene that encodes a DNA binding protein that specifically binds nuclear matrix attachment regions. A previous study disclosed the *SATB2* gene to be associated with tick burden, and the author mentioned a potential link of this gene to the immune system [56]. Jiang et al. [57] conducted a large-scale GWAS using first-lactation Holstein cows and identified the 91.13–94.62 MBp region of chromosome 5, including the *EPS8* gene associated with fat yield, covered by SNP AX-106726354 located near *EPS8* in this study. The SNP AX-124381310 on chromosome 24 was close to the *GATA6* gene. The GATA gene family has six members (*GATA1–GATA6*) in vertebrates and plays a critical role in controlling the growth, differentiation and survival of diverse cells, as well as maintaining the body’s functions [58]. Currently, mutations in *GATA6* were reported to be associated with microtia in Awassi sheep in ruminants [59].

Regarding the *COL12A1* (Collagen Type XII α 1 Chain) gene, it is known that degradation of the extracellular matrix and collagen chain trimerization is common among the related pathways of this gene. The Gene Ontology annotations associated with this gene include extracellular matrix structural constituent conferring tensile strength. Collagens consist of 28 members in vertebrates, and they are the most abundant extracellular matrix proteins [60]. Chen et al. [61] showed that gene expression levels of a collagen member (*COL4A1*) were significantly downregulated in the inflammation-associated fibroblasts from bovine mammary glands compared to normal fibroblasts, suggesting an implication of collagen members in immune response since one of the related pathways include extracellular matrix–receptor interaction. Moreover, Lu et al. [60] revealed that several collagen genes in bovine had higher expression levels during lactation, among which *COL12A1* is upregulated in late lactation, suggesting that the collagen genes had varied biological functions at different phases of lactation. In the present study, we identified two significant SNPs associated with SCS (rs43585636 and rs43585209) located close to *COL12A1*. All of the aforementioned aspects suggest that the *COL12A1* gene might be involved in SCS.

This study revealed that different markers and candidate genes were found for the two breeds in our study. In addition, all identified SNPs have been distinct among the three parities, denoting that SCS is influenced by different genes according to parity. The different sets of markers discovered in this work compared to studies reported in the literature can be attributed to various factors. We can assume that the power of detecting SNPs can be lower in the RB breed compared to the RS breed as a consequence of the comparatively lower number of genotyped RB animals used in the analysis. Furthermore, previously known mastitis related genes such as *LUZP2*, *AKAP8* and *MEGF10* were also identified in our study as candidate genes for SCS in the RB breed. For this specific case in the RB breed, further studies using larger sample sizes should be performed in order to validate previous results. Finally, an additional factor that may influence the different sets of candidate SNPs/breed is the distinct genetic backgrounds of the two breeds as they have different levels of clustering, as shown in previous studies on Romanian cattle breeds [62,63].

The specific novel SNPs and candidate genes located around them reported in the present study can be considered as candidates involved in SCS and mastitis resistance; however, these need to be kept in perspective, and polymorphisms in those genes should be further analyzed to highlight whether they influence the ability of dairy cows to resist mastitis.

## 5. Conclusions

Our study represents the first GWAS for SCS in Romanian dairy cattle and, thus, provides new perspective into the genetic architecture of udder infections in these native dairy cattle. We identified 41 SNPs and detected their significant associations with variation in SCS in dairy cattle. In both breeds, the SNPs and position of association signals were distinct among the three parities, denoting that mastitis is influenced by different genes according to parity. The results contribute to an increase in knowledge regarding the proportion of genetic variability explained by SNPs for SCS in dairy cattle and, particularly, in Romanian native cattle. However, further large-scale studies of a vast number of native dairy cattle breeds are needed in order to investigate others markers and genes that could be involved in mastitis.

## Figures and Tables

**Table 1:** Descriptive statistics for somatic cell score (SCS) in Romanian dairy cattle.

Breed	Trait	N	Mean	SD	Min	Max	*h^2^*	*V_P_*	*V_A_*
RS	LSCS_1_	11,081	2.81	1.72	−1.64	8.84	0.08	2.9916	0.2564
	LSCS_2_	7479	3.06	1.89	−2.06	8.82	0.10	3.4975	0.3686
	LSCS_3_	5735	3.27	1.88	−2.06	8.83	0.11	3.5347	0.3966
RB	LSCS_1_	3462	4.53	1.92	−0.84	8.82	0.03	2.7739	0.0857
	LSCS_2_	3072	4.51	1.86	−1.36	8.84	0.07	2.9304	0.2067
	LSCS_3_	2501	4.62	1.87	−1.64	8.82	0.06	3.0061	0.2048

RS, Romanian Spotted breed; RB, Romanian Brown breed; LSCS_1 to 3_, lactation average of somatic cell scores for 1st to 3rd lactations; N, number of test-day records; SD, standard deviation; Min, minimum score; Max, maximum score; *h*^2^, heritability; *V_P_*, phenotypic variance; *V_A_*, additive genetic variance.

**Table 2:** List of single nucleotide polymorphisms (SNPs) in the *Bos taurus* autosomes showing significant (−log_10_ (*p*) ≥ 4.00) associations with SCS in the Romanian Spotted breed.

Parity ^1^	Informative SNP ^2^	SNP rsID	Chr:Position ^3^	A1	A2	SNP Effect ^4^	–log_10_ (*p*)	Nearest Gene ^5^	Distance (bp)
L_1_									
	AX-115117070	NA	13:78383148	G	A	−4.74 × 10^−8^	4.28	NA	NA
	AX-117085597	rs137805472	1:95119031	C	T	5.34 × 10^−8^	4.07	NA	NA
L_2_									
	AX-106721594	NA	1:71370844	T	C	3.07 × 10^−8^	4.87	*ZDHHC19*	25909
	AX-106755404	NA	23:17013312	A	G	2.21 × 10^−8^	4.61	NA	NA
	AX-106740205	rs109232438	9:71884731	C	T	5.01 × 10^−8^	4.32	NA	NA
	AX-171465786	rs110140732	17:3030264	G	A	9.27 × 10^−8^	4.28	NA	NA
	AX-117088706	rs43209122	16:70728849	A	G	−2.50 × 10^−8^	4.23	NA	NA
	AX-106761299	NA	8:25435054	C	T	−1.70 × 10^−8^	4.23	NA	NA
L_3_									
	AX-106761943	rs110749552	6:37526622	G	A	5.47 × 10^−8^	6.37	*HERC3*	within
	AX-106728871	rs29021886	14:17674401	C	A	−1.20 × 10^−8^	4.62	NA	NA
	AX-124381671	NA	4:79254806	A	G	1.20 × 10^−8^	4.6	NA	NA
	AX-106740778	rs42627158	8:81747455	C	T	−7.00 × 10^−8^	4.36	*DAPK1*	459784
	AX-115112140	NA	15:5927942	A	G	3.73 × 10^−8^	4.19	*MMP7*	462217
	AX-185121504	rs209378984	6:70369168	A	G	5.97 × 10^−8^	4.11	NA	NA
	AX-115108867	NA	14:41169239	A	G	6.76 × 10^−9^	4	NA	NA

^1^ Parity 1 (L_1_), 2 (L_2_) and 3 (L_3_). ^2^ SNPs are sorted in descending order with respect to the significance of the association analysis between each SNP and SCS. ^3^ SNP position based on the UMD3.1 genome assembly of *Bos taurus.* ^4^ SNP effect is calculated relative to the A2 allele (if the effect is positive, then A2 increases the phenotype relative to A1) ^5^ Nearest gene: genes downstream/upstream relative to the SNP, within a ±1 MBp range (2 MBp total). NA: not available; SNP: single nucleotide polymorphism; Chr: chromosome; A1 and A2: allele 1 and 2.

**Table 3:** List of SNPs in the *Bos taurus* autosomes showing significant (−log_10_ (*p*) ≥ 4.00) associations with SCS in the Romanian Brown breed.

Parity ^1^	Informative SNP ^2^	SNP rsID	Chr:Position ^3^	A1	A2	SNP Effect ^4^	–log_10_ (*p*)	Nearest Gene ^5^	Distance (bp)
L_1_									
	AX-106741653	NA	7:8272794	C	A	6.14 × 10^−9^	5.37	*AKAP8*	514640
	AX-115114947	NA	7:8276425	G	A	6.14 × 10^−9^	5.37	*AKAP8*	511009
	AX-106739297	rs108991944	11:37261133	A	C	1.54 × 10^−9^	4.41	*CLHC1*	526855
	AX-124375018	rs42894728	6:20276795	G	A	7.39 × 10^−9^	4.33	NA	NA
	AX-171466608	rs42228650	7:27601185	G	A	5.65 × 10^−10^	4.29	*MEGF10*	219169
	AX-106727011	NA	13:76008898	G	A	−3.96 × 10^−9^	4.24	NA	NA
	AX-117090125	rs110642171	6:39688028	A	G	−2.65 × 10^−9^	4.19	NA	NA
	AX-117084655	rs109189476	2:88162563	C	T	6.31 × 10^−10^	4.14	*SATB2*	86169
	AX-106731475	NA	8:75798051	T	C	2.67 × 10^−9^	4.14	NA	NA
	AX-124381310	NA	24:34453446	A	G	−1.98 × 10^−9^	4.08	*GATA6*	96211
L_2_									
	AX-124381305	rs43358795	3:97560648	A	G	−3 × 10^−10^	5.38	*SPATA6*	809734
	AX-169513997	rs133252983	13:77447650	G	T	5.07 × 10^−9^	4.61	NA	NA
	AX-117080179	rs110709131	2:46155190	G	A	3.47 × 10^−9^	4.56	NA	NA
	AX-117083483	rs133489631	2:46126141	T	C	3.47 × 10^−9^	4.56	NA	NA
	AX-124350324	rs42797639	9:20734430	C	T	−9.3 × 10^−10^	4.19	NA	NA
	AX-124377880	rs41981703	21:40381539	A	C	1.02 × 10^−8^	4.15	NA	NA
	AX-106735825	rs43585636	9:14745626	C	A	3.02 × 10^−9^	4.09	*COL12A1*	123884
	AX-117085949	rs43585209	9:14767890	A	G	3.02 × 10^−9^	4.09	*COL12A1*	101620
	AX-106726354	NA	5:94517415	A	G	5.58 × 10^−9^	4.08	*EPS8*	156339
L_3_									
	AX-115109333	NA	22:26294371	A	G	6.59 × 10^−9^	5.17	NA	NA
	AX-124375014	rs110955314	29:19740081	C	A	−1.57 × 10^−8^	4.38	*LUZP2*	519687
	AX-106753982	NA	21:23675656	A	G	−6.09 × 10^−9^	4.35	*RAMAC*	30303
	AX-106735391	rs110359025	19:16700029	C	T	−7.19 × 10^−9^	4.21	NA	NA
	AX-106735614	rs43256975	1:108154057	A	G	−3.37 × 10^−9^	4.1	*IL12A*	131225
	AX-115115579	rs110825674	19:50547082	T	C	−9.06 × 10^−9^	4.06	NA	NA
	AX-106730586	rs109619272	20:22823334	A	G	4.48 × 10^−9^	4.03	*ANKRD55*	184056

^1^ Parity 1 (L_1_), 2 (L_2_) and 3 (L_3_). ^2^ SNPs are sorted in descending order with respect to the significance of the association analysis between each SNP and SCS. ^3^ SNP position based on the UMD3.1 genome assembly of *Bos taurus.* ^4^ SNP effect is calculated relative to the A2 allele (if the effect is positive, then A2 increases the phenotype relative to A1). ^5^ Nearest gene: genes downstream/upstream relative to the SNP, within a ±1 MBp range (2 MBp total). NA: not available; SNP: single nucleotide polymorphism; Chr: chromosome; A1 and A2: allele 1 and 2.

## Data Availability

Data will be available upon request.

## Supplementary Materials

The following are available online at https://www.mdpi.com/article/10.3390/genes12101495/s1, Figure S1: First 20 principal component analysis (PCA) of Romanian Spotted (RS) and Romanian Brown (RB) cattle. Figure S2: Heritabilities (in red) and repeatabilities (in blue) of somatic cell scores among parities (L1–L3) for Romanian Spotted (RS) and Romanian Brown (RB) cattle.
