# Supplementary material for: Genome-Wide Association Studies for Milk Somatic Cell Score in Romanian Dairy Cattle
Source: Genes (Basel). 2021 Sep 24;12(10):1495. doi: 10.3390/genes12101495 (PMC8535694; doi:10.3390/genes12101495)
Supplement: Supplementary file 1 [file genes-12-01495-s001.zip › genes-1384978-supplementary.pdf]

genes

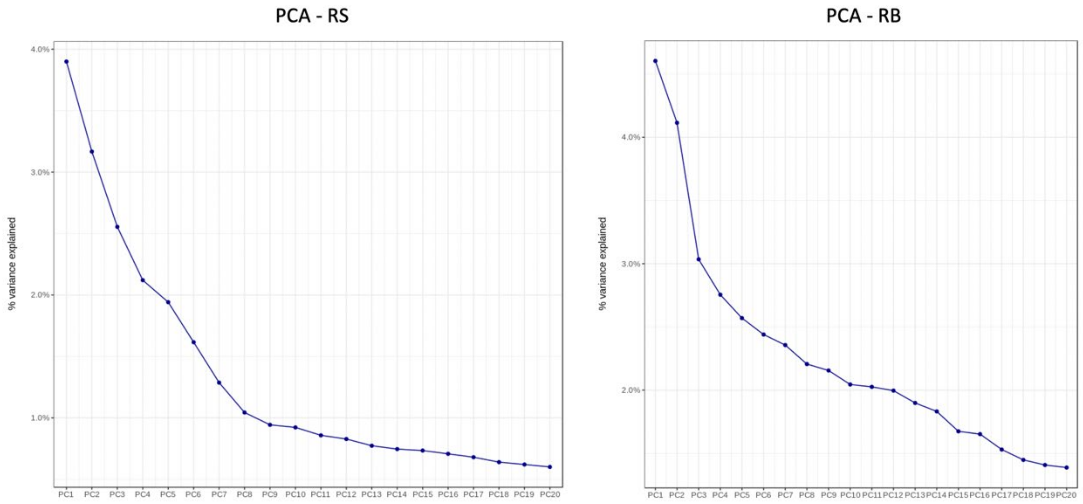

Figure S1. First 20 principal component analysis (PCA) of the Romanian Spotted (RS) and Romanian Brown (RB) cattle

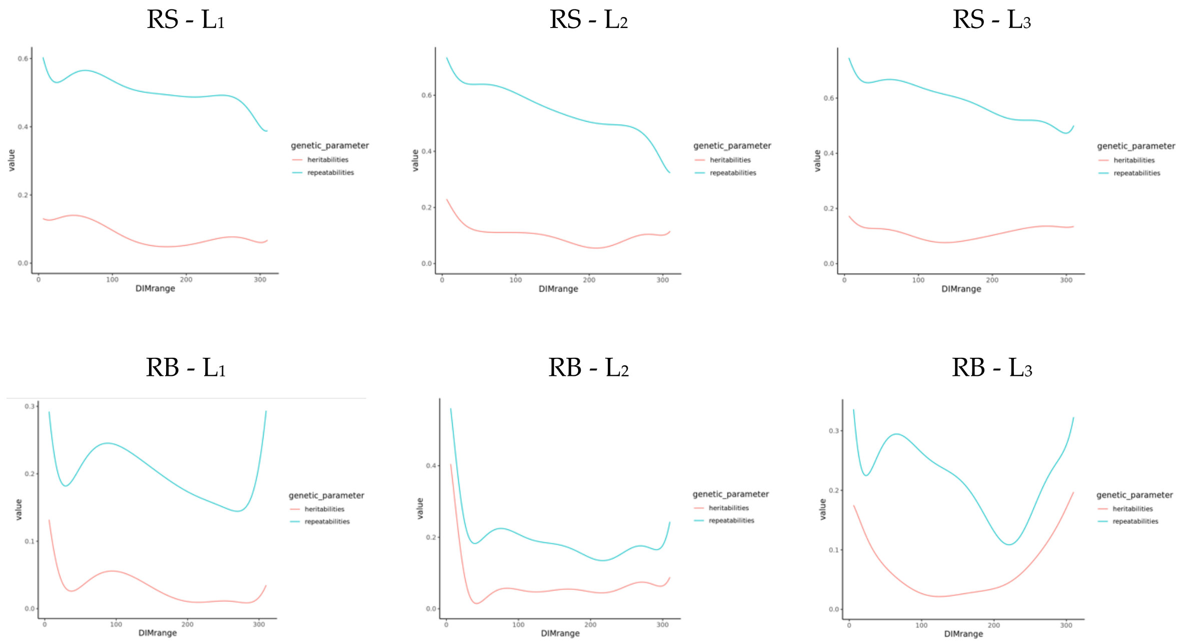

Figure S2. Heritabilities (in red) and repeatabilities (in blue) of somatic cell score among parities (L_1-L_3) for Romanian Spotted (RS) and Romanian Brown (RB) cattle.
